# Hypokalemic Quadriparesis Associated With Renal Glycosuria in Dengue Fever: A Rare Presentation

**DOI:** 10.7759/cureus.65489

**Published:** 2024-07-27

**Authors:** Jay Kakadiya, Kush Varsadiya, Chintan Kakadiya, Maulik Prajapati, Pritesh Patel

**Affiliations:** 1 Department of Internal Medicine, Government Medical College, Surat, Surat, IND; 2 Department of Internal Medicine, Shri M. P. (Meghaji Pethraj) Shah Government Medical College, Jamnagar, IND; 3 Department of Internal Medicine, Surat Municipal Institute of Medical Education and Research, Surat, IND

**Keywords:** proximal convulated tubule damage, quadriparesis, dengue, renal glycosuria, hypokalemia

## Abstract

Dengue fever is a viral hemorrhagic fever mainly transmitted by *Aedes *mosquitoes and is especially prevalent in equatorial regions. The presentation of dengue fever can range from mild symptoms, such as fever and body aches, to severe symptoms, such as hemorrhagic bleeding and shock. Although it is a non-neurotropic virus, it rarely manifests as a neurological abnormality. Previous data suggests that the incidence of electrolyte disturbance is increasing in patients with dengue. Here, we have described a case of dengue fever with hypokalemia and renal glycosuria. Studies show that the probable mechanism of developing hypokalemia is increased insulin and catecholamine, but it is still not well-established. We propose a mechanism that can explain both hypokalemia and renal glycosuria in our case.

## Introduction

Dengue fever is one of the most common arthropod-borne viral hemorrhagic fevers, which is especially prevalent in tropical and subtropical regions. It has a wide range of presentations from only simple self-resolving fever to hemorrhagic fever. Major clinical manifestations include fever, arthralgia, rash, and petechiae. It is a non-neurotropic virus, and neurological abnormalities are usually not seen with it; however, it rarely manifests as motor weakness or paralysis [1]. Previous data suggest that the chances of electrolyte disturbances in patients with dengue fever are very rapidly increasing, affecting nearly 30%-40% of cases, but a definite mechanism for this has not yet been identified [2].

## Case presentation

A 35-year-old male presented to the emergency with a complaint of weakness in all four limbs, which had started just one day ago. He had a history of fever and generalized body aches that started before three days and resolved after taking paracetamol. Weakness started from the lower limb and ascended upward to involve the upper limb. The patient has no history of diarrhea or ingestion of heavy carbohydrate meals. There was no prior history of such an episode and also no family history of the same condition. The patient did not have a history of diabetes mellitus (DM), asthma, major surgery, or blood transfusion. The patient has been consuming country liquor of approximately 150 ml/day for the past eight years and has been chewing tobacco and smoking for the past 12 years. On examination, the patient was vitally stable. He had a power of 3/5 in both upper limbs and 2/5 in both lower limbs. There was no sensory deficit or any evidence of bladder, bowel, or bulbar involvement.

On investigation, the peripheral smear showed macro-ovalocytes, and no malarial parasite was present. Laboratory parameters showed low platelet count, low serum potassium level, and high urine sugar level with normal blood sugar. The patient also has a low bicarbonate level, indicating metabolic acidosis. The laboratory values are mentioned in Table 1.

**Table 1:** Laboratory parameters HCO_3_-: Bicarbonate.

	Day 1	Day 2	Day 3
Hemoglobin (g/dL)	9.6	9.4	9.4
Red blood cells	2.47 x 10^6^/uL	2.4 x 10^6^/uL	2.6 x 10^6^/uL
White blood cells	10.1 x 10^3^/uL	8.2 x 10^3^/uL	5.2 x 10^3^/uL
Platelets	105 x 10^3^/uL	61 x 10^3^/uL	93 x 10^3^/uL
Serum potassium (mmol/L)	1.8	3.7	4.8
Serum sodium (mmol/L)	138	140	135
Serum magnesium (mmol/L)	1.1	-	-
Serum chloride (mmol/L)	101	-	-
Serum calcium (mmol/L)	2.5	-	-
Creatinine (mg/dL)	1.1	1.3	0.9
Alanine transferase (U/L)	74	-	-
Aspartate transferase (U/L)	133	-	-
Random blood sugar (mg/dl)	116	128	120
Urine sugar	3+	1+	Trace
Urinary pH	7.1	6.5	5.8
HCO_3_- (mEq/L)	11	14	20

**Figure 1:**
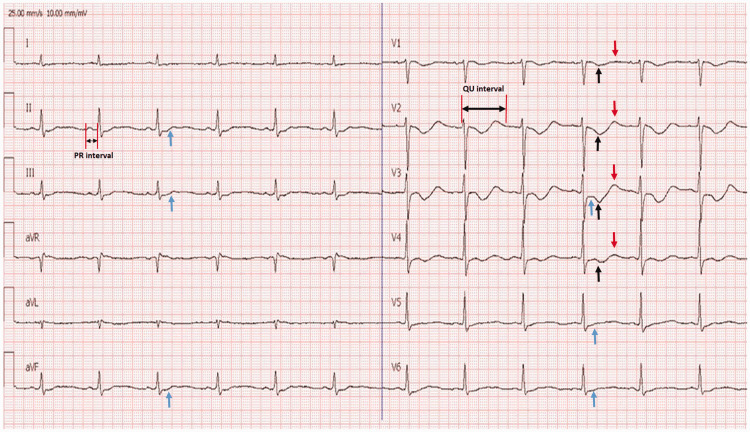
Hypokalemic changes in ECG The blue arrow indicates ST depression; the black arrow indicates T wave inversion, and the red arrow indicates prominent U waves.

By looking at the laboratory values, we suspected dengue fever and conducted tests for NS1, IgM, and IgG using the enzyme-linked immunosorbent assay (ELISA) method. NS1 and IgM were found to be positive, while IgG was negative. The ECG also supported hypokalemic presentation, showing mild ST depression, T wave inversion, and prominent U wave (Figure 1). A diagnosis of dengue fever with hypokalemic paralysis was made through clinical and laboratory evaluation.

The differential diagnosis of motor weakness in this case can be Guillain-Barré syndrome (GBS), familial hypokalemic periodic paralysis, and myositis. GBS was excluded by the nerve conduction velocity (NCV) study and the absence of a history of diarrheal illness. Familial hypokalemic periodic paralysis was excluded as there was no history of heavy carbohydrate ingestion and no similar history in any family member. Myositis can be eliminated as there was no pain with the weakness.

The patient was treated with I/V potassium chloride infusion at a rate of 10 mEq/hour, fluid therapy, and symptomatic measures. His blood potassium level improved to 4.8 mmol/L, and he regained normal motor power in all limbs. He was discharged on the fourth day of admission with 5/5 power in all limbs, normal electrolytes, and advice for follow-up.

## Discussion

Neurological manifestations are not common in dengue fever. However, dengue can rarely present with GBS, familial hypokalemic periodic paralysis, and myositis. Santos et al. [3] reported GBS associated with dengue fever. Gupta et al. [4] reported two cases of hypokalemic periodic paralysis. Kalita et al. [5] reported seven patients of myositis with dengue. Roy et al. [6] reported two confirmed cases of hypokalemic paralysis with dengue fever, which improved after potassium chloride (KCl) infusion. Our case has similarities with these studies. However, one unique aspect of our case is normoglycemia with renal glycosuria.

The mechanism of hypokalemia is not yet confirmed. Tomar et al. [7] proposed possible mechanisms for hypokalemia, either due to transcellular shift of potassium by increased insulin level or renal potassium wasting by increased catecholamine in stress. In our case, metabolic acidosis, urine sugar, hypokalemia, and alkaline urine were observed. Therefore, we propose that hypokalemia may be due to transient damage in epithelial cells of the proximal convoluted tubule (PCT) of the nephron caused by antigens or antibodies formed against the dengue virus [8]. This leads to an abnormality in the absorption of sodium and glucose as well as increased tubular sodium and glucose concentration.

This ultimately results in step-up reabsorption of sodium through the epithelial sodium channel (ENaC) in the principal cell of the nephron's collecting duct. To maintain the electrochemical gradient, potassium is secreted in the renal tubule through the renal outer medullary potassium channel (ROMK), thereby declining the serum potassium level. Therefore, the patient is presented with renal glycosuria and hypokalemia. As HCO_3_- is also wasted in the urine, the pH of urine is also increased. By looking at the laboratory values in our case, we propose that the possible mechanism of renal glycosuria and hypokalemia is transient PCT epithelial cell damage.

## Conclusions

This case report highlights urinary and neurological abnormalities in patients with dengue fever and the probable mechanism. Further research is needed in this sector to confirm these findings. Dengue virus can also lead to renal failure, and evidence supporting this is also established. Therefore, healthcare workers should pay special attention to patients presenting with motor weakness in the endemic area. Intravenous infusion of potassium is a lifeline for patients with hypokalemia, which leads to full recovery. As dengue also affects the renal system in some cases, creatinine and urine routine examinations should be conducted to prevent complications that can lead to morbidities and mortalities. Overall, our study provides valuable insights into the presentation and management of dengue fever.
